# The RPOC long axis is a simple indicator for predicting the need of invasive strategies for secondary postpartum hemorrhage in either post-abortion or post-partum women: a retrospective case control study

**DOI:** 10.1186/s12884-021-04083-y

**Published:** 2021-09-24

**Authors:** Mariya Kobayashi, Satoshi Nakagawa, Yoko Kawanishi, Tatsuo Masuda, Takahide Maenaka, Aska Toda, Tatsuya Miyake, Kosuke Hiramatsu, Ai Miyoshi, Kazuya Mimura, Toshihiro Kimura, Masayuki Endo, Tadashi Kimura

**Affiliations:** Department of Obstetrics and Gynecology, Osaka University Graduate School of Medicine, 2-2 Yamadaoka, Suita, Osaka, 565-0871 Japan

**Keywords:** Retained products of conception (RPOC), Secondary postpartum hemorrhage (sPPH), Retained placenta, Placental polyp, Arteriovenous malformation (AVM), Conservative management

## Abstract

**Background:**

The retained products of conception (RPOC) and related conditions (RPOC-ARC) are the main cause of secondary postpartum hemorrhage (sPPH), but there is no clear consensus for their management. The purpose of this study was to characterize those RPOC-ARC that require invasive treatment and those that could be managed more conservatively.

**Methods:**

We retrospectively analyzed 96 cases of RPOC-ARC that occurred after miscarriage, abortion, or delivery at a gestational age between 12 and 42 completed weeks, that were managed within our institution from May 2015 to August 2020. We reviewed the associations between the occurrence of sPPH requiring invasive treatment with clinical factors such as the maternal background and the characteristics of the lesions.

**Results:**

The range of gestational age at delivery in our study was 12–21 weeks in 61 cases, 22–36 in 5, and 37 or later in 30. Among them, nine cases required invasive procedures for treatment. The onset of sPPH was within one month of delivery in all but two cases, with a median of 24 days (range 9–47). We found significant differences between requirements for invasive versus non-invasive strategies according to gestational age at delivery, assisted reproductive technology (ART) pregnancy, amount of blood loss at delivery, and the long axis of the RPOC-ARC lesion (*p* = 0.028, *p* = 0.009, *p* = 0.004, and *p* = 0.002, respectively). Multivariate analysis showed that only the long axis of the lesion showed a significant difference (*p* = 0.029). The Receiver Operating Characteristic (ROC) curve for predicting the need for invasive strategies using the long axis of the lesion showed that with a cutoff of 4.4 cm, the sensitivity, specificity, positive predictive value (PPV), and negative predictive value (NPV) was 87.5, 90.0, 43.8, and 98.7%, respectively.

**Conclusion:**

The long axis of the RPOC-ARC is a simple indicator for predicting which sPPH will require invasive procedures, which use is rare in cases with lesions less than 4.4 cm or those occurring after the first postpartum month. Conservative management should be considered in such cases.

## Background

Postpartum hemorrhage (PPH) is an important cause of maternal mortality. There are two types of PPH, which are dependent on the time of hemorrhage onset. The first is primary PPH (pPPH) which occurs in the first 24 h following delivery, the other is the secondary PPH (sPPH) that occurs from 24 h or up to 12 weeks postpartum [1]. The presence of a uterine mass, with or without vascularity, after a miscarriage, abortion, or delivery is a key cause of sPPH. The retained products of conception (RPOC) refers to any intrauterine tissue of trophoblastic origin that is present or that develops after delivery. It was reported that 33.3% of sPPH is due to such RPOC [2].

An arteriovenous malformation (AVM) refers to a vascular anomaly where arteries and veins are directly connected through a complex void of the usual intermediate capillary bed. AVM is reported to account for only 3.3% of sPPH [2], making sPPH due to AVM very rare, compared to RPOC. Recently, AVM has sometimes been described as ‘enhanced myometrial vascularity’ (EMV), which refers to a focus of abnormally increased vascularity in the post-partum myometrium. Although RPOC, AVM, and other post-partum lesions are pathologically classified as different conditions, in practice it is difficult to accurately differentiate them clinically, and they sometimes coexist. Furthermore, since RPOC with vascularity and AVM often require a similar treatment approach, there might be little clinical significance for a strict differentiation. Therefore, we refer to these vascular abnormalities as ‘retained products of conception and related conditions (RPOC-ARC)’.

PPH is the leading cause of maternal mortality worldwide, accounting for 1/3 to 1/4 of all maternal deaths [3], and the incidence of PPH is reported to be increasing [4]. Guidelines for predicting the onset of pPPH and for its management have been provided in various countries; however, there is as yet no established management strategy for sPPH. Various reports have proposed conservative management for these conditions versus aggressive management strategies, such as curettage, operative hysteroscopy, uterine artery embolization (UAE), and hysterectomy, but no clear consensus treatment strategy has been established. We believe that clarifying the predictors of sPPH development will be useful in establishing an evidence-based management plan for RPOC-ARC. In this study, we have investigated the outcome of clinically suspected ‘RPOC-ARC’ in our institution, in order to determine the risk factors for sPPH requiring invasive strategies.

## Methods

This study included patients who experienced miscarriage, abortion, or delivery at a gestational age between 12 and 42 completed weeks between May 2015 and August 2020. In Japan, pregnancies after 12 weeks of gestation are routinely delivered via vaginal delivery or cesarean section, not by dilatation and evacuation and/or curettage. Cases of placenta previa were excluded from our study. Patients were managed for sPPH at our institution, the Osaka University Hospital, after they had completed their delivery at our institution or elsewhere. In our institution, invasive strategies for RPOC-ARC are performed only in cases of developing sPPH, which is defined as excessive bleeding that occurs more than 24 h after delivery and up to 12 weeks postpartum. Only one case, where a prophylactic hysterectomy was performed according to the patient’s request, was excluded from our study.

As routine care at our institution, clinicians perform an initial two-dimensional transvaginal ultrasonography (TvUS) 1–5 days after delivery (at the time of discharge), and an additional TvUS is conducted one month after discharge. In addition, TvUS was routinely evaluated at 1–2 weeks postpartum in cases of delivery at 12–21 weeks of gestation. When patients came to our hospital urgently due to vaginal bleeding, imaging studies, including TvUS, transabdominal ultrasonography, computed tomography (CT), and/or magnetic resonance image (MRI), were performed, as needed. Among patients whose pregnancies were terminated at other hospitals, those whose puerperium were managed in the same way as at our hospital, and those who completed their treatment at our hospital, were included in this study. All cases of suspected abnormalities were followed until the lesions disappeared.

Electronic medical records were searched for the terms “PROC”, “AVM”, “EMV”, and their synonyms (e.g., placental retention and placental polyp) in English and Japanese. M.K. (lead author) reviewed the records for maternal age, the institution of delivery, gravidity (including this most recent conception), parity(including this conception), the use of assisted reproductive technology (ART), the gestational age at delivery, the mode of delivery, the amount of blood loss at delivery (including the amount of amniotic fluid), whether manual removal of the placenta (MRP) was performed at delivery, the long axis of the ‘RPOC-ARC’, and the clinical management and the outcome of the sPPH. As for the long axis of the lesion, the longest axis detected was adopted. Since delivery of the placenta is often challenging in patients with early delivery due to placental immaturity, so whether MRP was or was not performed was examined only in patients with a delivery after 37 weeks of gestation.

To compare the maternal backgrounds, including information regarding the ‘RPOC-ARC’, and the need for invasive strategies due to the development of sPPH, univariate analysis was performed using a logistic regression analysis for continuous variables and the Chi-square test for nominal variables. In addition, multivariate analysis was performed using logistic analysis for all parameters that were statistically significant in the univariate analysis. All analyses were performed using JMP® Pro 15 (SAS Institute Inc., Cary, NC, USA), and *p* < 0.05 was considered statistically significant.

## Results

A total of 96 patients were included in this study: 74 patients who delivered at our institution and 22 patients who delivered at other institutions. Table 1 shows the patients’ characteristics and information about their RPOC-ARC. The median period from delivery to the use of an invasive strategy due to sPPH was 24 days (range 9–47), and the onset was within 1 month in all but two cases. Of the cases in which an invasive strategy was performed, six had hemostasis conducted by UAE, and three had a hysterectomy (two of these three had to have UAE performed for a primary PPH).

**Table 1:** Patient’s characteristics and information about RPOC-ARC

	*n*=96
Maternal age (years), median (range)	36 (20-46)
< 30, n (%)	11 (11.5)
30 - 34, n (%)	27 (28.1)
35 - 39, n (%)	39 (40.6)
40 ≦, n (%)	19 (19.8)
Institution of delivery	
Our institution	74 (77.1)
Other institutions	22 (22.9)
Primipara, n (%)	45 (46.9)
ART pregnancy, n(%) *1	30 (31.3)
Gestational age at delivery, median (range)	21 (12-41)
< 30 weeks, n (%)	61 (63.4)
30+0 - 36+6weeks, n (%)	5 (6.2)
37 weeks≦, n (%)	30 (31.3)
Mode of delivery	
Vaginal, n (%)	88 (91.7)
CS, n (%)	8 (8.3)
Blood loss at delivery (ml), median (range) *2	459 (24-4654)
MPR at delievry, n(%) *3	13 (43.3)
Long axis of the lesion (cm), median (range) *4	3.0 (0.7-11.6)
< 4cm, n(%)	75 (78.1)
4cm ≦, n(%)	20 (20.1)
**Intervention required, n (%)**	9 (9.4)
Regimen of intervention, n (%) *5	
UAE, n (%)	6 (6.3)
Hysterectomy, n (%)	3 (3.1)
During between delivery and intervention (days), median (range) *5	24 (9-47)
< 31 days, n (%)	7 (77.8)
31 days ≦, n (%)	2 (22.2)

*1:Six cases were missing data. *2:Six cases were missing data. *3:Only cases of delivery after 37 weeks of gestation. *4:One case was missing data. *5:Of the nine cases that required intervention

Table 2 shows the univariate analysis of the predictors of clinical management and the outcome of the RPOC-ARC. A statistically significant difference was observed in the gestational age at delivery (*p* = 0.028), ART pregnancy (*p* = 0.009), blood loss at delivery (*p* = 0.004), and the longest axis of the lesion (*p* = 0.002). There was no significant difference in whether or not MRP was performed among the cases after 37 weeks (*p* = 0.723, odds ratio (OR) =1.333, 95% CI: 0.252–7.118).

**Table 2:** Univariate analysis of predictors of clinical outcome of RPOC-ARC

	Invasive strategy				
	(+) n=9	(-) n=88	Odds ratio	95% CI	*p* value
Maternal age (years), median (range)	38 (29-45)	36 (20-46)	1.074	0.930, 1.240	0.333
Gravidity	2 (1-4)	2 (1-5)	1.130	0.606, 2.105	0.700
Parity	2 (1-2)	2 (1-4)	1.167	0.384, 3.014	0.763
Gestational age at delivery, median (range)	39 (36-41)	20 (12-41)	1.126	1.025, 1.538	0.028
ART pregnancy, n(%)	6 (66.7)	24 (27.6)	7.250	1.365, 38.494	0.009
Mode of delivery, n(%)	1 (11.1) *1	7 (8.0) *1	1.429	0.156, 13.124	0.751
Blood loss at delivery (ml), median (range)	1330 (586-4556)	320 (24-4654)	1.001	1.000, 1.001	0.004
Long axis of the lesion (cm), median (range)	5.3 (3.8-9.0)	2.7 (0.7-11.6)	1.555	1.179, 2.049	0.002

*1: n (%) of cesarean section

**Fig. 1:**
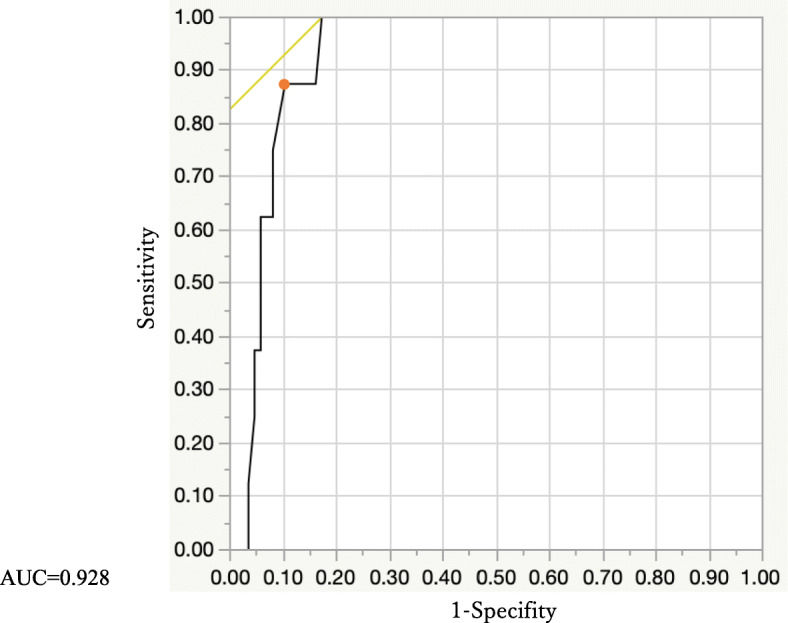
ROC curve for predicting the need for invasive strategies using the long axis of RPOC-ARC. AUC = 0.928. With a cutoff of 4.4 cm for the long axis of the lesion (solid circle), the sensitivity, specificity, PPV and NPV were 87.5, 90.0, 43.8 and 98.7%, respectively

Table 3 shows the result of the multivariate analysis of the parameters that were statistically significant in univariate analysis. Only the longest axis of the lesion showed a significant difference (*p* = 0.029). Therefore, we prepared a receiver-operating characteristic (ROC) curve for predicting the need for invasive strategies using the long axis of the RPOC-ARC (AUC = 0.928) (Fig. 1). With a cutoff of 4.4 cm for the long axis of the lesion, the sensitivity, specificity, positive predictive value (PPV), and negative predictive value (NPV) were 87.5, 90.0, 43.8, and 98.7%, respectively.

**Table 3:** Result of the multivariate analysis of parameters that were statistically significant in univariate analysis

	Odds ratio	95% CI	*p* value
Gestational age at delivery, median (range)	1.249	0.894, 1.744	0.193
ART pregnancy, n (%)	16.641	0.676, 409.573	0.085
Blood loss at delivery (ml), median (range)	1.000	0.999, 1.001	0.909
Long axis of the lesion (cm), median (range)	1.743	1.058, 2.872	0.029

## Discussion

This study resulted in three important findings: 1) sPPH due to ‘RPOC-ARC’ only rarely occurs after the first month after delivery; 2) The long axis of the lesion is helpful in predicting the development of sPPH requiring invasive treatment; 3) When the long axis cut-off is set to 4.4 cm, the NPV is about 98.7%, which means that lesions smaller than 4.4 cm can be safely managed conservatively.

We found that ultrasound is the most used imaging method in the diagnosis of these RPOC-ARC. A systematic review to evaluate postpartum ultrasound for the diagnosis of RPOC concludes that it should be suspected with a thickened endometrial echo complex with a cut-off value of 10 mm or the presence of an intracavitary mass, and that the detection of hypervascularity, with color Doppler ultrasound, in addition to these findings, is very sensitive for RPOC [5]. If these features are not visible, RPOC is rare. CT and MRI can help in the diagnosis of RPOC, but they are not used as the first line of investigation since these methods are far less convenient than ultrasound. Sonohysterography (SHG) was reported to have a greater accuracy than ultrasound in the detection of RPOC, but after SHG, 17.9% of cases showed complications, such as fever and infection [6]. Therefore, SHG is not recommended because of these adverse effects.

A previous retrospective study from our institution regarding the conservative management of RPOC-ARC reported that, of the 319 medical abortions at 12–21 weeks of gestation, 75 (24%) had a sonographically-identified EMV, but all these EMV resolved spontaneously, regardless of symptoms, and none with an associated massive bleeding required an invasive treatment [7]. On the other hand, another retrospective study reported that of their 59 RPOCs, including 40 (68%) labors after term, 36 (61%) required interventions due to bleeding-related events, such as significant bleeding (20/36) or continuous small amounts of bleeding (16/36) [8]. These results suggest that conservative management for RPOC-ARC is effective, but that the need for aggressive intervention increases as the number of gestational weeks of delivery advances.

Surgical managements, such as dilation-and-curettage or hysteroscopic resection, are known. Operative hysteroscopy for RPOC several months after delivery has been reported to have a high efficacy rate and yields a subsequent high retention of fertility (the efficacy rate: 91%, the subsequent fertility among those who desire another pregnancy: 83%) [9]. Compared to conventional curettage, hysteroscopic resection is reported to have a higher rate of complete resection, fewer complications such as perforation and adhesions, a better rate of subsequent pregnancy, and a shorter time to subsequent pregnancy [10, 11]. Therefore, hysteroscopy should be the choice, rather than curettage. However, these procedures tend to be avoided in the case of RPOC with high blood flow soon after delivery due to the fear of bleeding during the procedure.

A previous retrospective study reported that the selective UAE is effective, without incidents or post-embolization complications, with a 74.2% (23/31) success rate in the primary UAE, and a combined success rate of 87.1% (27/31) in the primary and second UAE [12]. Another report showed that the technical and clinical success rate of UAE using a gelatin sponge for RPOC with hemorrhage was achieved without major complications in 93 and 100% of such cases, respectively [13]. These suggest that UAE may be useful for RPOC with marked vascularity, AVM, or EMV for preventing the risk of hemorrhage related to the surgical removal of the mass with methods such as curettage or operative hysteroscopy. Three-dimensional (3D) color Doppler ultrasound, which is minimally invasive, may be useful in determining the reduction of blood flow [14]. In addition, prophylactic UAE used before obstetric procedures with a high risk for massive bleeding was shown to be a safe and effective [15]. A series of treatments, such as with UAE to reduce vascularity, 3D color Doppler evaluation, and subsequent hysteroscopic surgery might be reasonable procedures for the RPOC with high blood flow; however, no evidence-based methods for the management of ‘RPOC-ARC’ have been established to date, so this is an issue for further study and the indications for prophylactic procedures should be carefully evaluated.

In order to determine whether and when conservative management is likely to resolve RPOC-ARC, it is necessary to judge the risk of developing sPPH on a case by case basis. It was reported that patients with severe complications had a significantly larger axis of the lesion than those without serious complications [16]. A previous retrospective cohort study concluded that having a maximum length of the RPOC of ≧4 cm (adjusted OR = 8.6, 95% CI:2.4–39.2) and its hypervascularity (adjusted OR = 4.6, 95% CI:1.3–18.8) were independent risk factors for requiring intervention due to bleeding-related events [8]. Another retrospective study reported that serum free-hemoglobin, endometrial stripe thickness, and US vascularity score are significant predictors of the need for surgical intervention in women with clinically suspected RPOC [17]. In this study, we found that lesions smaller than 4.4 cm can be managed conservatively with relative safety. Previous reports support the results of our study, however, the greatest strength of our study is its ability to classify the risk of developing sPPH in a simpler way than previous reports.

The final decision regarding RPOC management should be made in discussion with the patient and her family, taking into account her living environments, such as her place of residence and the support and cooperation expected from her family, since emergency consultations will be necessary if sPPH develops during conservative treatment. Most importantly, clinicians must understand the various data reported about RPOC-ARC and must present them clearly to their patients and families.

Our study had some limitations. First, this study was a retrospective case-control study. Therefore, the type and timing of examinations were decided by each clinician, and we could not examine the optimal timing and methods for the detection of the RPOC-ARC. Second, histopathological confirmation was not possible, an unavoidable limitation, since most of the cases were treated conservatively or using UAE. Third, we examined various subgroups combined: post-abortion at 12–21 weeks of gestation, post-miscarriage at 12–21 weeks of gestation, and post-partum delivery at 22 weeks of gestation or later. We tried to examine these separately, but could not find significant results in each subgroup (data not shown). We consider that this may be attributable to a lack of power. The present study showed that invasive procedures for RPOC-ARC were required more frequently in post-partum cases after 37 weeks of gestation, 26.7% (8/30). Therefore, we expect to research only such cases. To carry this out, further investigation with a numerous sample is necessary, and this is an issue for future study. Even with these limitations, this study revealed that the risk of sPPH requiring invasive strategies related to RPOC-ARC may be accurately assessed by evaluating solely the long axis of the lesion. More research is needed to obtain a high level of evidence for preventing sPPH due to RPOC-ARC.

## Conclusions

When considering the management of RPOC-ARC, it is important to be familiar with the risk factors of sPPH. The long axis of the lesion is a simple and useful indicator for predicting sPPH requiring invasive treatment, as sPPH due to RPOC-ARC is rare in cases less where the long axis of the lesion is less than 4.4 cm. Conservative management of the lesion can be considered in such cases. For lesions larger than 4.4 cm, it is necessary to present a careful management plan tailored to each patients’ background and condition.

## Data Availability

The datasets used and/or analyzed during the current study are available from the corresponding author on reasonable request.

## Abbreviations

RPOC: Retained products of conception
RPOC-ARC: RPOC and related conditions
PPH: Postpartum hemorrhage
sPPH: Secondary PPH
pPPH: Primary PPH
ART: Assisted reproductive technology
AVM: Arteriovenous; malformation
EMV: Enhanced myometrial vascularity
UAE: Uterine artery embolization
TvUS: Two-dimensional transvaginal ultrasonography
CT: Computed Tomography
MRI: Magnetic resonance image
MRP: Manual removal of placenta
OR: Odds ratio
ROC: Receiver operating characteristic
PPV: Positive predictive value
NPV: Negative predictive value
SHG: Sonohysterography
3D: Three-dimensional
